# Characterization of Omental Immune Aggregates during Establishment of a Latent Gammaherpesvirus Infection

**DOI:** 10.1371/journal.pone.0043196

**Published:** 2012-08-29

**Authors:** Kathleen S. Gray, Christopher M. Collins, Samuel H. Speck

**Affiliations:** Emory Vaccine Center and Department of Microbiology and Immunology, Emory University School of Medicine, Atlanta, Georgia, United States of America; Karolinska Institutet, Sweden

## Abstract

Herpesviruses are characterized by their ability to establish lifelong latent infection. The gammaherpesvirus subfamily is distinguished by lymphotropism, establishing and maintaining latent infection predominantly in B lymphocytes. Consequently, gammaherpesvirus pathogenesis is closely linked to normal B cell physiology. Murine gammaherpesvirus 68 (MHV68) pathogenesis in laboratory mice has been extensively studied as a model system to gain insights into the nature of gammaherpesvirus infection in B cells and their associated lymphoid compartments. In addition to B cells, MHV68 infection of macrophages contributes significantly to the frequency of viral genome-positive cells in the peritoneal cavity throughout latency. The omentum, a sheet of richly-vascularized adipose tissue, resides in the peritoneal cavity and contains clusters of immune cell aggregates termed milky spots. Although the value of the omentum in surgical wound-healing has long been appreciated, the unique properties of this tissue and its contribution to both innate and adaptive immunity have only recently been recognized. To determine whether the omentum plays a role in gammaherpesvirus pathogenesis we examined this site during early MHV68 infection and long-term latency. Following intraperitoneal infection, immune aggregates within the omentum expanded in size and number and contained virus-infected cells. Notably, a germinal-center B cell population appeared in the omentum of infected animals with earlier kinetics and greater magnitude than that observed in the spleen. Furthermore, the omentum harbored a stable frequency of viral genome-positive cells through early and into long-term latency, while removal of the omentum prior to infection resulted in a slight decrease in the establishment of splenic latency following intraperitoneal infection. These data provide the first evidence that the omentum is a site of chronic MHV68 infection that may contribute to the maintenance of chronic infection.

## Introduction

Gammaherpesviruses are ubiquitous pathogens, with many viruses identified and exhibiting tropism across a variety of different species, including elephants, rodents, non-human primates, and humans. Among humans, Epstein-Barr virus (EBV) is the most common gammaherpesvirus with as many as 95% of humans tested exhibiting seropositivity. With the exception of EBV-related infectious mononucleosis, which is most often observed in adolescents, primary EBV infection is asymptomatic and is maintained without appreciable consequence in the majority of individuals. Kaposi's Sarcoma-associated herpesvirus (KSHV) is another less common but relatively widespread human gammaherpesvirus. Like EBV, KSHV is usually of limited pathogenecity in healthy, immunocompetent individuals. However, when the immune system is compromised, the presumably innocuous host-pathogen balance is disrupted and gammaherpesvirus-associated pathogeneses arise. The most common of these are lymphoproliferative diseases such as PTLD, often presenting in solid-organ transplant patients on intensive immunosuppression regimens, and PEL and Kaposi's sarcoma, seen most often in AIDS patients with profoundly compromised immune systems.

Lifelong gammaherpesvirus infection is thought to begin an acute phase in which viral replication and egress allows for the recruitment and infection of long-term latency targets, namely memory B cells. An alternative but not mutually-exclusive theory is that viral proteins in infected cells actually promote naïve-to-memory B cell differentiation such that the virus drives the primary B cell to the stage preferred for long-term latency (i.e., quiescent long-lived memory B cells). While the latency reservoir for alpha, beta, and gammaherpesviruses differs, this unique strategy is one that appears to be shared by all herpesvirus family members and allows infection to be maintained in relatively dormant cell types.

Many of the gammaherpesviruses, including the human pathogens EBV and KSHV, exhibit a very narrow host tropism. Thus, studies on EBV and KSHV have largely been limited to the confines of in vitro experimentation. The isolation of a murid gammaherpesvirus from wild bank voles was an important step forward in the deeper understanding of human gammaherpesviruses in that it enabled the detailed study of viral pathogenesis in the context of a natural host. Extensive evidence has since been generated to support the genetic, pathogenic, and immunogenic similarity of murine gammaherpesviruses to those of humans and non-human primates (reviewed in [1], [2]). Therefore, the MHV68 system has become a well-accepted model to study aspects of gammaherpesvirus pathogenesis.

One of the most important aspects supporting the relevance of the mouse model is the demonstration that, like EBV infection in humans, memory B cells are the major long-term reservoir of MHV68 latency [3]. As EBV and MHV68 genomes can be detected in these cells from the peripheral blood and spleen, respectively, it is of interest to examine the anatomical sites that serve as possible reservoirs or conduits for memory B cells and determine their relevance to gammaherpesvirus infection. While the spleen and lymph nodes are comprised of mainly cells of hematopoietic origin, sites with mixed tissue composition have also been shown to be important for the transmission and long-term maintenance of both EBV and KSHV. In humans, for example, the tonsils are composed of lymphocytes and plasma cells interspersed among epithelial cells, and several groups have demonstrated gammaherpesvirus infection or the presence of gammaherpesvirus-positive cells in the lung, Peyer's-patch-containing intestinal epithelium, and salivary glands of mice [4], [5], [6], [7]. In addition to lymphocytes, these structures contain a combination of endothelial/mesothelial/myeloid-derived cells and exhibit unique characteristics in terms of responses to pathogens, inflammation, and hormones, all of which have been shown to mediate aspects of gammaherpesvirus infection. The microenvironment can distinctively shape aspects of infection by promoting or suppressing lytic replication thorough the direct action of cytokines or chemokines on infected cells or indirectly through the recruitment of specific cell populations.

The omentum is a structure within the peritoneal cavity comprised largely of adipose tissue and mesothelial cells. Distinct aggregates of lymphoid cells, termed “milky spots” due to their dense clustering and appearance, were first observed in the omenta of rabbits. Subsequent work has demonstrated that this tissue is a rich source of lymphocytes and monocytes, and possesses tremendous antimicrobial and angiogenic properties - hence its frequent use in surgical procedures to aide internal wound healing. It has also been shown that the fetal omentum is critical to the development of B1 B cells and may in fact play an important role in the homeostasis of this cell population in adult mice and humans [8], [9], [10]. Despite the presence of organized B and T-cell-containing structures within milky spots, the omentum has never been widely classified as a true secondary lymphoid organ. Although controversy still exists around its precise immunological definition, recent evidence has demonstrated the complex and sophisticated nature of the omentum and revealed its important role in both innate and adaptive immunity [11], [12]. Due to these unique immunological properties, we examined the role of the omentum following MHV68 infection and show here that it is both an early and latent reservoir for MHV68 following intraperitoneal infection. In addition, we show that the omentum appears to support germinal center formation as well as MHV68 infection of macrophages following i.p. infection and may therefore provide an environment to facilitate the interaction of these two cell types and contribute to the efficient establishment of splenic latency following intraperitoneal infection.

## Results

### Morphological changes in the omentum following MHV68 infection

**Figure 1:**
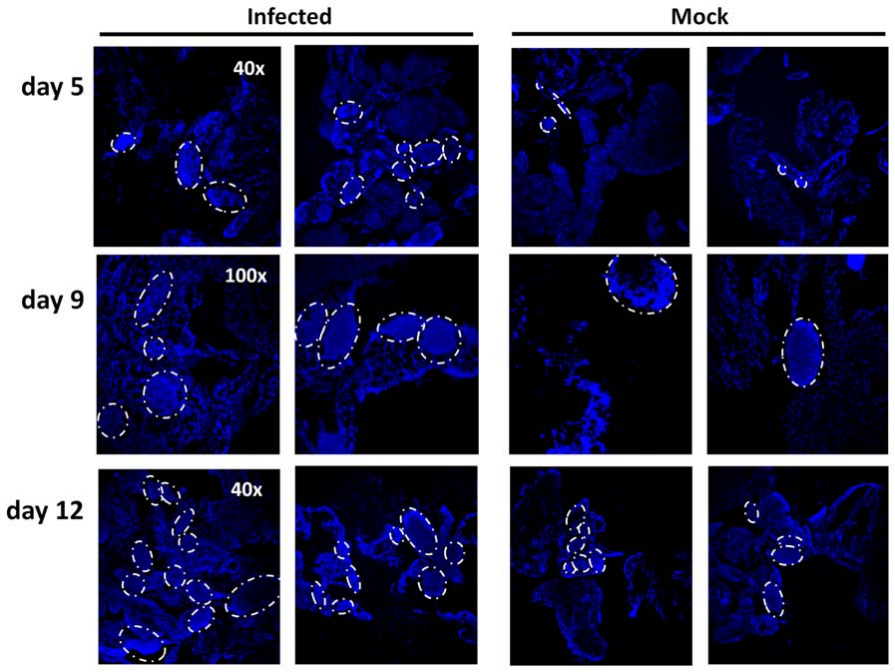
Increased size and frequency of omental immune aggregates in MHV68-infected mice. Omenta from mice inoculated intraperitoneally (i.p.) with 1000 plaque forming units (pfu) virus in complete media (cDMEM) or cDMEM alone (mock) and harvested at the indicated times post infection. Omenta were cryosectioned and stained using DAPI-containing mounting media. Dense DAPI-staining in immune aggregates, “milky spots,” is outlined by dashed ovals.

The mouse omentum has been shown to support rapid accumulation of tumor cells and antigen, exhibiting proliferative effects and morphological changes within hours of intraperitoneal injection [13], [14]. We therefore first examined the omentum for phenotypic alterations following intraperitoneal (i.p.) infection with a recombinant MHV68 virus expressing enhanced yellow fluorescent protein (eYFP), MHV68-H2bYFP [15]. Omental milky spots appear as dense nuclear aggregates amidst highly-vascularized adipose tissue. While no obvious morphological changes were observed at day 2 post infection, both the size and number of these dense nuclear regions was increased in the omentum of MHV68-infected versus mock-infected animals at day 5 post infection (Fig. 1). These aggregates were increased in size and distribution frequency at day 9 and more so at day 12 post-infection. Immunofluorescence and histochemistry studies have revealed that omental milky spots have distinct, germinal-center like properties, including FDC-M1+ cells and highly proliferative B-cell zones capable of supporting class-switching and somatic hypermutation in response to infection [12], [14]. Notably increased follicle size or frequency was not observed in the spleen by day 5 (data not shown), suggesting that peritoneal MHV68 infection induces a immunological response in the omentum which results in sustained expansion of germinal center-like lymphoid aggregates with distinct kinetics from that observed in the spleen.

### Expansion of germinal center cells following MHV68 infection

**Figure 2:**
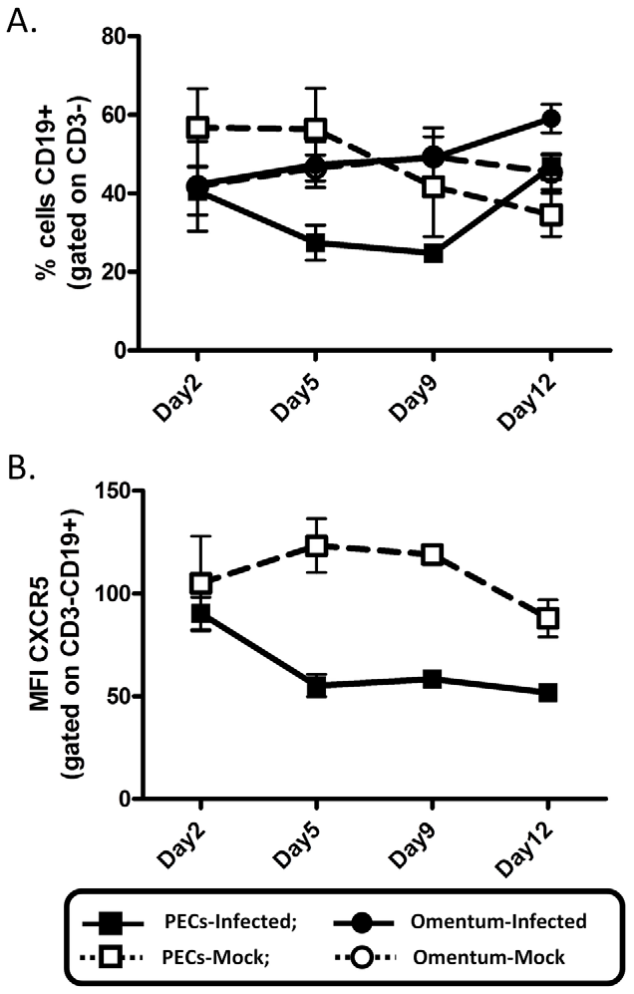
B cell populations in omentum and peritoneal exudate following MHV68 infection. Cells from the omentum and peritoneal cavity (PECs) were isolated at the indicated times post-intraperitoneal infection and analyzed by flow cytometry. Live cells were gated on A) CD3^−^ and total B cells determined by CD19 expression or B) CD3^−^CD19^+^ cells to determine CXCR5 expression.

MHV68 infection following intranasal and intraperitoneal infection has largely been characterized by examining the spleen and, to a lesser extent, cells from the peritoneal cavity [16], [17], [18], [19]. To define any unique changes in cell populations within the omentum following i.p. MHV68 infection, we examined cells isolated from the omentum and compared these populations to those found in splenocytes and peritoneal exudate cells (PECs). There were several notable changes in macrophage subsets in the peritoneum versus the spleen and omentum (data not shown), but the most distinct alterations were seen in populations of B cells. Upon MHV68 infection, total B cell frequencies in the peritoneal cavity were rapidly reduced by nearly half between day 2 and 5 and remained low before increasing between day 9 and 12 post-infection (Fig. 2, panels A & B). This may have been due to an efflux of B cells out of the peritoneal cavity and into adjacent lymphoid tissues such as the omentum or spleen, however B cell frequencies remain unchanged at these sites. Alternatively, this may reflect an influx of other cell types, such as T cell and/or macrophages, in response to the peritonitis induced by MHV68 infection.

**Figure 3:**
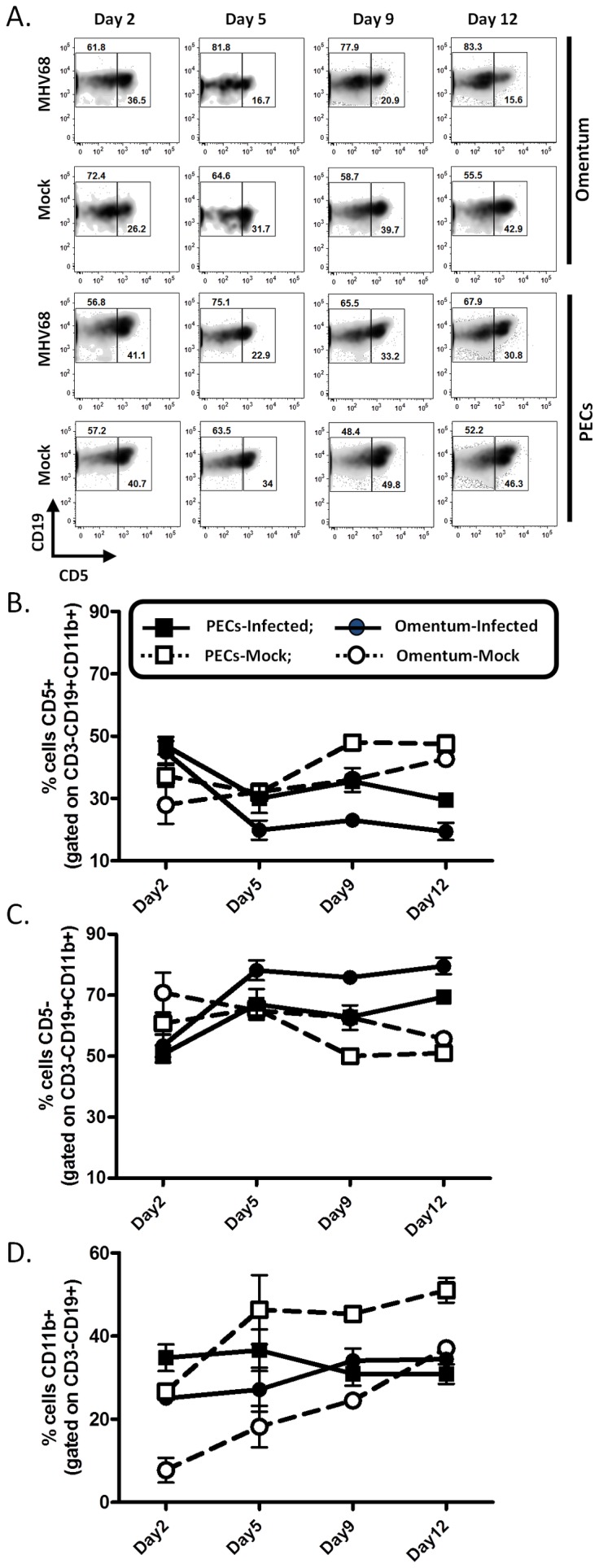
Alterations in B1-B cell populations in the omentum and peritoneal exudate following MHV68 infection. Cells from the omentum and peritoneal cavity (PECs) were isolated at the indicated times post-intraperitoneal infection and analyzed by flow cytometry. Live cells were gated on CD3^−^CD19^+^CD11b^+^ cells and B1-a and B1-b populations discriminated by CD5 expression. A) Representative flow plots and B–D) compiled data from experimental groups of mice represented as line graphs for B1a B cells (B), B1b B cells (C), and total B1 B cell (D) frequencies. Squares, [cells from] peritoneal cavity; Circles, [cells from] omentum; Open symbols, mock-infected; filled symbols, MHV68-infected.

The peritoneal cavity is an abundant source of B1 B cells, innate-like lymphocytes important in the response to peritoneal antigens and the primary source of “natural” IgM. Numerous studies have documented the response of B1 B cells to peritoneal antigens, noting that this population is exquisitely sensitive compared to B2 B cells and trafficks en masse out of the peritoneal cavity and to the omentum during infection. We therefore assessed B1 B cells and two B1 B cell subpopulations: B1-a (CD19^+^CD11b^+^CD5^+^) and B1-b cells (CD19^+^CD11b^+^CD5^−^). We observed that while the frequency of total B1 B cells remains relatively constant in infected animals, the frequency of B1-a cells decreased steadily in both the peritoneum and omentum while that of B1-b cells subsequently increased (Fig. 3). This pattern is distinct from the steady increase in total B1 B cells and relatively modest changes in B1a versus B1b cell frequency in mock-infected animals (Fig. 3D). We attribute changes in mock infected animals to the presence of fetal calf serum in the complete media used for inoculation. A recent study has also reported a similar observation, noting that B1-a B cells rapidly disappear from the peritoneal cavity following i.p. LPS injection and attributed this to chemokine-mediated migration toward CXCL12 and CXCL13 gradients - although only CXCR4 expression is increased on B1-a B cells while CXCR5 expression remains constant [20]. We observed a similar reduction in B1-a B cell frequency in PECs (Fig. 3B), yet the disappearance was more gradual and was preceded by a dramatic reduction in total B cell CXCR5 expression (Fig. 2B) These differences are not entirely surprising, as LPS-induced peritonitis is distinct from that induced by MHV68. In addition, the MHV68 M3 gene encodes a secreted protein that can bind and sequester the CXCR5 ligand, CXCL13, which may affect both cellular migration as well as CXCR5 expression on surrounding cells.

**Figure 4:**
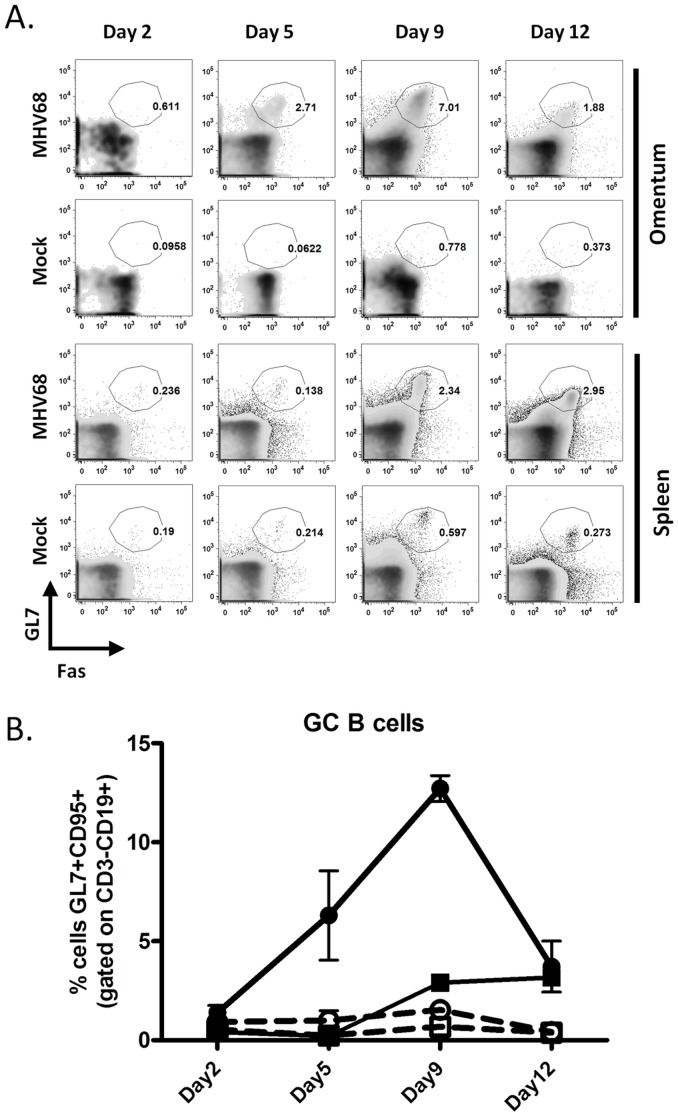
Germinal center B cell expansion in omentum precedes expansion in spleen. Cells from the omentum and spleen were isolated at the indicated times post-intraperitoneal infection and analyzed by flow cytometry. Live cells were gated on CD3^−^CD19^+^ cells and germinal center populations identified by Fas (CD95) and GL7 expression. A) Representative flow plots and B) data from individual mice represented as line graph. Squares, [cells from] spleen; Circles, [cells from] omentum; Open symbols, mock-infected; filled symbols, MHV68-infected.

A striking feature was the appearance of germinal center (GC)-phenotype (CD19^+^Fas^+^GL7^+^) cells in the omentum by day 5 post-infection, prior to the expansion of this population in the spleen (Fig. 4, panels A & B). The frequency of GC cells in the omentum further increased through day 9, at which point this population was detectable in the spleen. However, by day 12 post-infection the omental germinal center B cell population sharply decreased - while the levels of splenic germinal center B cells remained largely unchanged from days 9 to 12 (Fig. 4, panels A & B).

### Visualization of virus-infected cells in the omentum

**Figure 5:**
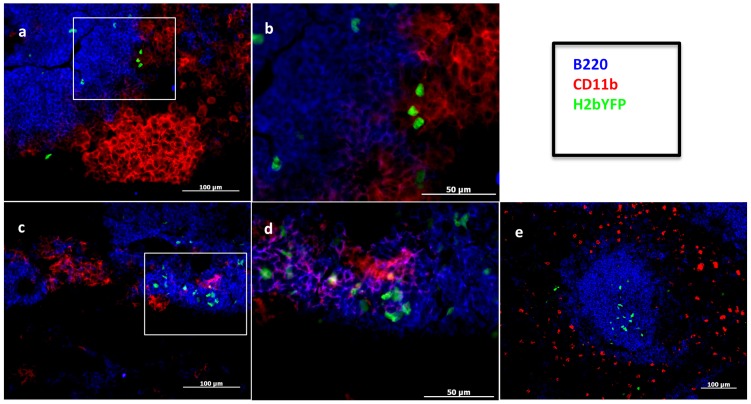
Visualization of YFP+ cells in the omentum. Omenta from mice inoculated intraperitoneally (i.p.) with 1000 plaque forming units (pfu) virus in complete media (cDMEM) or cDMEM alone (mock) and harvested at day 9 (omentum) and day 12 (spleen). Omenta (A–D) and spleen (E) were cryosectioned and stained with the antibodies to detect YFP, CD11b or B220 as described in Materials and Methods. a) YFP+ (virus-infected) cells within the B220+ area of omental immune aggregate; boxed area magnified in (b). c) CD11b+ YFP+ cells; boxed area magnified in (d). e) YFP+ splenic B cell follicles with surrounding CD11b+ extrafollicular region.

Using a recombinant MHV68 (MHV68-H2bYFP) that provides sustained expression of yellow fluorescent protein (YFP) in infected cells, we have recently demonstrated that the majority of YFP^+^ cells in the spleen at early latency time points (days 14–18) bear a germinal center phenotype and are found amongst proliferating B cells in splenic follicles [21]. Given the kinetics and magnitude of expansion of this population in the omentum, we also examined cells from the omentum of MHV68-H2bYFP infected mice to determine if CD19^+^Fas^+^GL7^+^ cells in the omentum also supported virus infection. Although YFP^+^ cells were detectable in the omentum by flow cytometry (data not shown), the frequency was lower than that seen in the spleen (see data and discussion in the following section). As such, we were unable to unambiguously phenotype the YFP+ population(s) using this approach. However, we were able to visualize YFP+ cells in the omentum by immunofluorescence using an antibody against the fluorescent protein. These analyses revealed virus-infected cells specifically within omental immune aggregates. Unlike splenic follicles, where virus-infected (YFP+) cells are primarily restricted to the B cell zone (Fig. 5E), we were able to detect YFP+ cells not only situated within the B220+ area of immune aggregates (Fig. 5, panels C & D), but also among CD11b+ cells outside of the B cell-rich center (Fig. 5, panels A & B). In addition, we also identified YFP+ cells that were not B220+ and were most likely virus infected CD11b+ macrophages (Fig. 5B). Notably, this is the first time YFP expression has been visualized in an MHV68 infected non-B cell population, suggesting that the populations of cells supporting virus infection in the omentum may be distinct from those in the spleen - perhaps more similar to MHV68 infection of the peritoneal cavity where virus infected macrophages represent the predominant infected cell population and only ca. 10% of the infected cells are B cells [18]. The localization of YFP+ cells in the interface of the B220+ and CD11b+ region may then raise the possibility that interactions between these types of cells are important in the early phases of MHV68 infection. Additionally, the CD11b+ cells within omental immune aggregates have been demonstrated to produce CXCL13. Given that CXCR5 ligand binding by CXC13 induces receptor internalization, it is possible that the decrease in CXCR5 expression on PECs, as well as cells within the omentum (data not shown), results from proximity to these CXCL13-producing CD11b+ cells.

### Omentum supports early latent infection and may contribute to seeding viral latency

**Figure 6:**
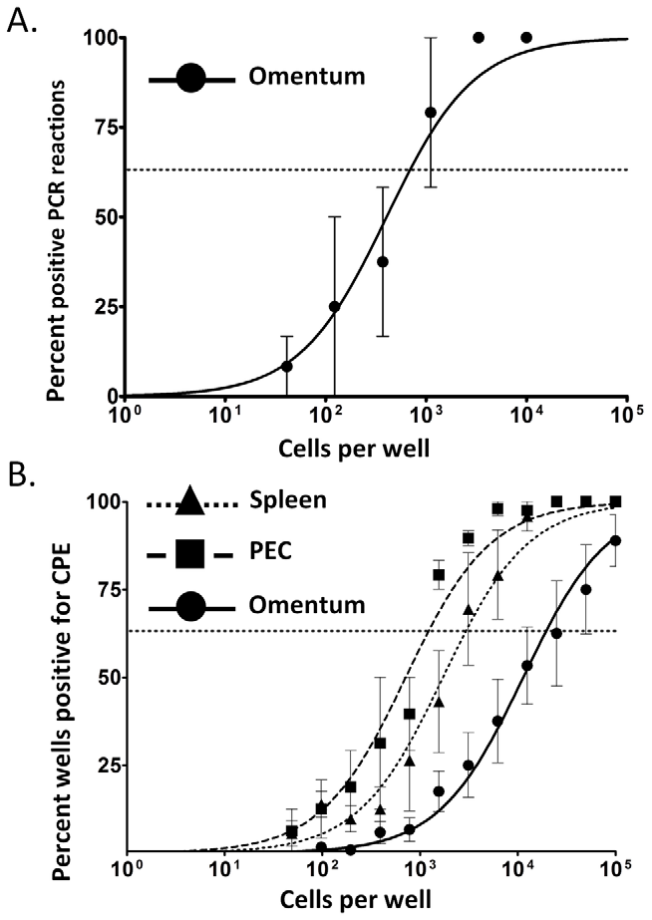
The omentum supports early latent infection. Mice were inoculated i.p. with 1000 pfu virus and PECs, spleen and omenta harvested at day 18 post-infection. Single cell suspensions were used for limiting dilution PCR analyses (omentum) (A) or prepared (see Materials and Methods) and plated immediately for limiting dilution reactivation analyses (spleen, peritoneum, and omentum) (B). A) Frequency of positive PCR reactions after limiting-dilution plating and DNA-quantity controlled PK digest/PCR. B) Frequency of CPE in MEF monolayers after limiting-dilution plating of intact cells as evidence of virus reactivation. Mechanically-disrupted cells were plated in parallel to control for the presence of preformed infectious virus (data not shown).

Given that macrophages and B cells support the majority of latent infection in the peritoneal cavity and spleen, we next assessed whether the omentum is also a reservoir for latent MHV68 following i.p inoculation of virus. We performed limiting-dilution PCR and ex vivo reactivation analyses to determine the frequency of viral genome-positive and reactivating cells, respectively, in the omentum versus the spleen and peritoneal cavity during early latency. At day 18 post-infection, the frequency of genome-positive cells in the omentum was about 1 in 700 (Fig. 6A), slightly lower than the frequency typically seen in splenocytes or PECs (about 1 in 100) at this time [17], [18], [22]. Likewise, the frequency of reactivating cells in the omentum was about 1 in 19,500 - lower that than observed in the spleen and peritoneal cavity (Fig. 6B). Notably, reactivation of infected cells from the omentum appeared to be less efficient than from either the spleen or PECs, as only about 4 percent of infected cells spontaneously reactivated virus in the limiting dilution analyses (compared to ∼10% of latently infected splenocytes and nearly 100 percent of latently infected PECs typically observed). This may result from cytokine-mediated suppression of lytic replication by particular cells plated along with virus-infected cells in the omentum, or by infected cells themselves. Regardless, these data again indicate that infection in the omentum has unique characteristics that appear to be distinct from MHV68 infection in the spleen or peritoneal cavity at day 18 post-infection. Finally, in parallel, mechanically disrupted cells were also plated in parallel to assess the presence of pre-formed infectious virus in the harvested omental tissue. Notably, no preformed infectious virus was detected in these lysates (data not shown), demonstrating that the omentum is a bona fide site of MHV68 latency following i.p. infection.

**Figure 7:**
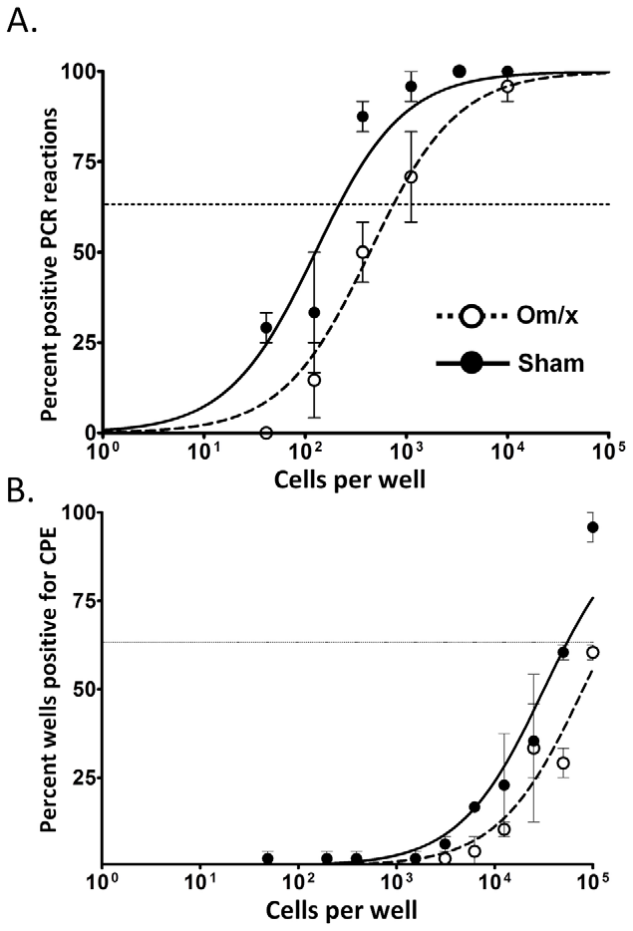
Reduced establishment in omentectomized mice during early latency. Mice underwent omentectomy or sham surgery and allowed to recover for four weeks, then were inoculated i.p. with 1000 pfu virus. Spleen and omenta were harvested at day 18 post-infection and single cell suspensions analyzed by limiting dilution reactivation analyses (A), or used for limiting dilution PCR analyses (B). A) Frequency of CPE in MEF monolayers after limiting-dilution plating of intact cells as evidence of virus reactivation. Mechanically-disrupted cells were plated in parallel to control for the presence of preformed infectious virus (data not shown). B) Frequency of positive PCR reactions after limiting-dilution plating and DNA-quantity-controlled PK digest/PCR.

The increased efficiency of reactivation of MHV68 from PECs versus splenocytes is thought to be accounted for by the predominance of infected macrophages in the peritoneal exudates. Macrophages support productive infection in vitro and therefore may be considered a more “active” environment favoring lytic replication (data not shown). Given the abundance of macrophages in omental immune aggregates, along with evidence suggesting that the omentum contains infected CD11b+ cells, we hypothesized that cells within the omentum may support virus replication and play a role in seeding viral latency - perhaps through productive virus replication and/or recruitment of naïve B cells- following i.p. infection. To test this hypothesis, we performed surgical resection of the omentum on mice four weeks prior to MHV68 infection to assess the effect of omentectomy on viral latency. Notably, control mice that underwent sham surgery and had intact omenta had a slightly higher frequency of viral genome-positive (1 in 220 versus 1 in 780) and reactivating cells (1 in 50,000 versus 1 in approximately 110,000) at day 18 following i.p. infection than omentectomized mice (Fig. 7). Although the observed decrease in latency is modest, the data are consistent with the omentum contributing to the efficient establishment of splenic latency by providing an environment conducive to lytic infection (much like the lungs following intranasal infection) and/or by recruiting lymphocytes (both B and T cells) to immune aggregates early during infection. Unfortunately, attempts to measure lytic virus in the omentum by plaque assay were confounded by cytotoxicity to the fibroblast monolayer - perhaps due to adipocytes in the omentum homogenate. Regardless, given the data presented here demonstrating virus-positive cells in the omentum, coupled with the earlier appearance of germinal center cells relative to the spleen, leaves open the possibility that the unique cellular heterogeneity of the omentum contributes to seeding of splenic latency by both lytic replication and chemokine-mediated recruitment of naïve B cells following i.p infection.

### The omentum supports long-term MHV68 infection

**Figure 8:**
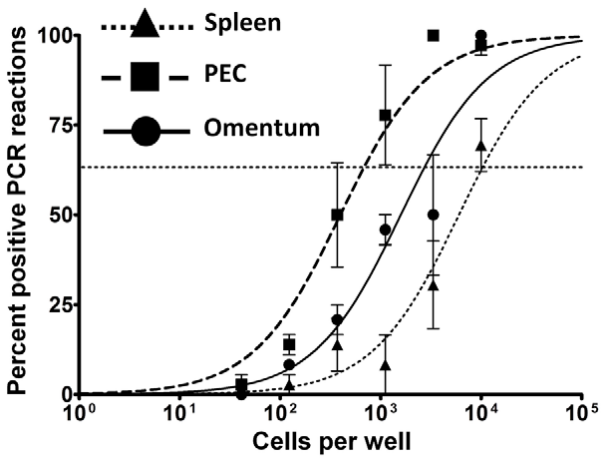
The omentum harbors MHV68 infected cells during long-term latency. Mice were inoculated i.p. with 1000 pfu virus and PECs, spleen, and omenta harvested at day 90 post-infection. Single cell suspensions were prepared used for limiting dilution PCR analyses to determine the frequency of viral genome positive cells.

The frequency of both viral genome-positive and reactivating cells is higher in PECs than splenocytes, both during early latency and at later times post-infection. While the frequency of viral genome-positive splenocytes decreases >10-fold between 3 and 12 weeks post-infection, PECs experience a less dramatic contraction of latently infected cells. This may be due in part to the nature of infection in the major virus reservoir within each compartment - while the majority of infected cells in the spleen are IgD-negative memory B cells at day 90, macrophages still comprise the bulk of infected cells in the peritoneum [18]. Since macrophages appear to be more conducive to lytic replication than B cells, it is possible that this process facilitates the infection of naïve cells and the maintenance of viral loads in macrophage-rich compartments. In support of this, viral genome loads in the omentum were similar at day 18 and day 90, and higher than those in the spleen at day 90 (Fig. 8; also see Fig. 6). One possible explanation is that the omentum maintains infection in PECs by providing a cellular niche for lytic replication and/or reactivation. Another possibility is that infected cells from the peritoneum collect in the omentum or traverse through this tissue during exit to the periphery. The dynamics of this remain to be elucidated, but based on the observations presented here the omentum appears to represent a site of chronic carriage of viral genome-positive cells following i.p. inoculation of virus. In addition to macrophage and lymphocyte populations in the lung, this may provide further opportunity to examine the interaction of these cell populations at a unique anatomical site.

## Discussion

The omentum has previously been identified as a tissue supporting early MHV68 infection [23]. Our studies confirm these observations and further characterize the nature of this infection, identifying virus-positive cells within immune aggregates in the omentum following i.p. infection. Furthermore, we demonstrate a marked immunological response to MHV68 infection in the omentum by this route, including growth and expansion of immune aggregates and a germinal center population that occurs prior to a similar response in the spleen. We extend the characterization to latent infection and demonstrate viral genome-positive cells in the omentum during early latency and the maintenance of these cells in long-term latency.

The importance of the omentum in surgical wound healing has long been recognized and its unique characteristics have received renewed interest from an immunological perspective [12], [24]. Its contribution to the development and maintenance of B-1 B cells is a subject of recent interest, as are its angiogenic and chemoattractant properties. The omentum is highly vascularized and has been shown to contain hypoxic regions within the immune aggregates/milky spots. These cells produce abundant levels of vascular endothelial growth factor (VEGF) and other pro-angiogenesis chemokines that are believed to account for the complex lymphatic and capillary networks feeding omental tissue. This may be of relevance to gammaherpesvirus pathogenesis, as several lines of evidence have demonstrated that hypoxia and one of its primary associated proteins, hypoxia inducible factor (HIF), activate gene50/Rta/BRLF1 transcription and induce reactivation from latency [25], [26]. In addition, evidence is emerging to further define the role of these proteins in macrophage effector functions - these HIF-mediated cellular processes may also be of importance in shaping aspects of macrophage infection within these immune aggregates [27], [28].

In addition to VEGF, omental cells are also potent producers of chemokines, particularly CXCL13. This chemokine is important not only for B1-B cell development and normal lymphocyte trafficking to the spleen and lymph nodes, but has also been shown to be important for lymphocyte flux between the periphery and the peritoneal cavity [11]. This cellular movement is thought to be mediated in part by various interactions between lymphocytes and cells within the omentum, including chemokines/receptors and integrins/addressins [12], [29], [30] – which at least partially dictate the flow of cells between peripheral circulation and that within central body cavities and may be of relevance to gammaherpesvirus pathogenesis. Moon et al recently demonstrated that B1a B cells are much more responsive to CXCL12/13 after LPS treatment and migrate out of the peritoneal cavity [20]. We also observed a reduction of peritoneal B1a, but not B1b, B cells, as well as a precipitous decrease in the numbers of peritoneal macrophages. MHV68 genome-positive cells are found in the peritoneal cavity at day 42 post-infection at the same frequency following intranasal or intraperitoneal inoculation, suggesting that infected lymphocytes and macrophages eventually complete a circuit that brings them from the blood and into the peritoneal cavity. If cells pass through the omentum during this process, they are exposed to the unique physiological properties of that tissue. Interestingly, many gammaherpesvirus-associated lymphomas and proliferative diseases, such as primary effusion lymphoma (PEL), manifest in body cavities. Although no direct involvement of the omentum in these pathologies has been defined, the identification of viral genome-positive cells in the omentum during lytic, early-latent, and long-term latent infection provides a basis for further consideration of its role. Although intraperitoneal infection is not a physiological or natural route of infection, the ability of the omentum to support MHV68 with distinct characteristics during early and long-term latency suggests that this tissue is not an inert, static reservoir of MHV68-infected cells but a dynamic site of MHV68 infection, much like secondary lymphoid-tissues. We have also assessed the presence of MHV68-positive cells in omentum following intranasal infection. A significant number of viral genome-positive cells were found at d. 50 post-infection (data not shown), suggesting that MHV68-infected cells aggregate in the omentum during chronic MHV68 infection, regardless of the route-of-inoculation. The expedient formation of germinal centers and appearance of MHV68-positive cells in the omentum soon after direct exposure to MHV68 via peritoneal infection also reflects an environment conducive to antigen-induced lymphocyte proliferation. This may be especially relevant for omental flap placement (where omental tissue is resected and used to patch surgical wounds) at various anatomical sites, including immunopriveleged sites like the surface of the brain.

The data presented here underscores the growing appreciation that MHV68 infection involves a complex anatomic profile and is most likely affected by every type of tissue into which infected cells come into contact. Along these lines, the normal flux of lymphocytes most likely influences several aspects of viral infection, including virus trafficking and reactivation. It is clear now that this is not restricted to secondary lymph node structures like the spleen and lymph nodes, but also less-studied structures like the omentum and mucosal tissues. These structures, particularly the omentum, are of distinct cellular composition, rich in non-lymphoid cells like monocytes, adipocytes, and cells of endothelial or mesothelial origin. Interestingly, omental milky spots develop in lymphotoxin-alpha (LT-α)-deficient mice while the development of organized secondary lymphoid structures like the spleen and lymph nodes are compromised [12]. Despite these splenic and lymph node deficiencies, LT-α deficient mice still class-switch following MHV68 infection and establish latency [31]. With the ability to support germinal center formation upon MHV68 infection demonstrated here, it may therefore be possible that germinal center B cells in the omentum play a role in the establishment of gammaherpesvirus latency.

## Materials and Methods

### Ethics Statement

This study was carried out in strict accordance with the recommendations in the Guide for the Care and Use of Laboratory Animals of the National Institutes of Health. The protocol was approved by the Emory University Institutional Animal Care and Use Committee and in accordance with established guidelines and policies at Emory University School of Medicine (Protocol no. 046-2010). All inoculations and surgery were performed under isoflurane anesthesia followed by post-surgical analgesic treatment, and all efforts were taken to minimize suffering.

### Mice

C57BL6/J mice (Jackson Laboratories, Bar Harbor, ME) aged 6–12 weeks were used for all experiments. For omentectomy experiments, mice were 6–8 weeks old upon surgery and 10–12 weeks old upon viral infection.

### Virus and Infection

For flow cytometry and immunofluorescence experiments, mice were infected with MHV68-Ins-H2B-YFP recombinant virus, described and characterized previously [15]. Virus was diluted in complete Dulbecco's Modified Eagle's Medium supplemented (cDMEM) with 10% fetal calf serum to a final concentration of 5000 pfu/ml. Mice were anesthetized briefly by isoflurane inhalation and inoculated intraperitoneally with 200 µl of diluted virus or cDMEM as a control (mock). For experiments to assess latent infection (genome loads and reactivation), mice were infected intraperitoneally with wild-type MHV68 WUMS strain (ATCC VR-1465) as above.

### Organ harvest and cell preparation

Mice were sacrificed by prolonged isoflurane inhalation and cervical dislocation. All cells and organs were placed on ice immediately upon retrieval. Peritoneal exudate cells (PECs) were collected by peritoneal lavage using 10 mL cDMEM. PECs were retrieved by centrifugation and resuspension in the appropriate media or buffer. For flow cytometry and latency analysis individual spleens were mechanically disrupted using mesh screens and glass pestles and homogenates centrifuged. Cell pellets underwent 1–2 red blood cell lysis treatments using Tris-Ammonium-Chloride buffer (Sigma) before centrifugation and resuspension of red-blood-cell depleted cell pellets in the appropriate media or buffer. Individual omenta were excised from surrounding adipose or pancreatic tissue and incubated for one hour at 37 degrees in 0.5% collagenase IV in BSA with intermittent vortexing. Digested omenta were then homogenized as above and resuspended in cDMEM. Adipocytes were allowed to collect at the media surface for 15 minutes at room temperature before under-layer containing adipocytes-depleted omental cells was transferred to a separate tube for centrifugation and resuspension in the appropriate media or buffer. For immunofluorescence analysis spleens and omenta were removed as above and stored on ice. Whole organs were placed into cryomolds and overlayed with O.C.T. (optimal cutting temperature) media (TissueTek) and placed into isobutane on a dry-ice bed. Upon solidification of the O.C.T media, cryomolds were stored at −70 degrees C. Four to 6-µm sections were prepared using a cryostat with internal temperature set to −30 degrees C. Sections were placed onto positively-charged glass slides and allowed to dry at room temperature for 24 hours before storage at −70 degrees C until staining.

### Immunofluorescence

Slides containing tissue sections were warmed to room temperature prior to staining. Tissues were rehydrated by incubation in phosphate-buffered saline (PBS) for 10 minutes at room temperature with gentle rocking. Tissues were first blocked using PBS containing 3% BSA/0.1% Tween, then stained with a master mix containing fluorochrome-conjugated antibodies (FITC-Anti-GFP (Rockland Immunochemicals for Research), PE-Anti-CD11b (clone M1/70, BD Pharmingen, and APC-Anti CD45/B220 (clone RA3-6B2, BD Pharmingen)) for 45 minutes at room temperature and protected from light. Staining solution was removed by aspiration and slides washed twice in PBS for 2 minutes per wash at room temperature with gentle rocking. Excess PBS was removed and sections overlayed with ProLong Gold Antifade reagent containing DAPI (Invitrogen) before cover slide placement. Sections were immediately visualized or stored at 4 degrees protected from light for up to two days. Images were captured using a Zeiss fluorescence microscope as described previously [15].

### Flow cytometry

Single cell suspensions were prepared as above. One to two million cells from peritoneal exudate, spleen, or omentum of individual mice were aliquoted into 96-well plates and stained as previously described [21], [22]. Antibodies used for flow cytometry were PE-Anti-F4/80 (clone BM8, eBioscience), PerCP-Anti-CD5 (clone 53-7.3, BD Pharmingen), PeCy7-Anti-CD11b (clone M1/70, eBioscience), APC-Anti-CXCR4 (clone 2B11, eBioscience), APC-Anti-Cy7-CD19 (clone 1D3, eBioscience), PacBlue-Anti- CD3 (clone UCHT1, BD Pharmingen), PE-Anti-CXCR5 (clone 2G8, BD Pharmingen), PE-Cy7-Anti-CD95 (clone Jo8, BD Pharmingen), and Biotin-Anti-GL7 with APC-streptavidin (Invitrogen). Stained cells were immediately analyzed on an LSR II flow cytometer using FACS Diva acquisition software. Analysis was performed using FlowJo software (Treestar Inc, San Carlos, CA) and graphs and statistical analyses (unpaired t-test) generated using GraphPad Prism software Version 5.0 (GraphPad Software, San Diego, CA).

### Latency analyses

Limiting dilution reactivation and genome analyses were performed as described previously [3], [17], [18]. Briefly, single-cell suspensions from each organ were pooled from individual mice and used for ex vivo and PCR-based analyses. To assess reactivation, cells were plated in cDMEM starting at 100,000cells per well and with 2-fold dilutions serial dilution onto 96-well plates containing mouse embryonic fibroblast (MEF) monolayers (two rows per dilution). Plates were incubated for 3 weeks and reactivation assessed by quantifying wells exhibiting cytopathic effect (CPE) as a result of virus reactivation. The presence of preformed infectious virus was controlled for by parallel plating of mechanically-disrupted cells. To assess genome frequency, intact cells were plated in 3-fold serial dilutions into wells of 96-well plate containing a background of 3T12 fibroblasts to maintain consistent DNA quantities per well and copy-number control reactions. Following proteinase-K digestion, nested PCR was performed and products visualized by gel electrophoresis. Graphs were generated using GraphPad Prism software and frequency of reactivating or genome-positive cells determined by Poisson distribution as previously described.

### Surgical procedures

Mice were anesthetized using 4% isoflurane in an isolated chamber and maintained under 1% isoflurane anesthetization for the duration of the procedure. The abdominal area was prepared by hair removal and disinfection with iodine and isopropyl alcohol swabs. A small incision was made on the right abdominal side to expose the peritoneum. Another incision was made in the peritoneal lining; the exposed omentum was drawn out from the peritoneum using sterile forceps and removed using a cautery pen. Remaining adipose tissue was placed back into the peritoneal cavity and the incisions repaired by double-wound closure using Vicryl non-braided suture (Ethicon). For sham surgeries, the omentum was isolated and drawn out as above but replaced into the peritoneal cavity without cauterization. Mice were removed from anesthetization and allowed to recover on cages positioned partially on heated water pads. Upon revival, mice were administered an oral analgesic (Metacam) and again on days 2 and 3 following surgery. Mice were monitored for signs of infection or distress before virus infection 4 weeks upon completion of surgery.
